# Influence of Body Mass Index, Physical Fitness, and Physical Activity on Energy Expenditure during Recess

**DOI:** 10.3390/children11010125

**Published:** 2024-01-18

**Authors:** Carlos Ayán-Pérez, Daniel González-Devesa, José Carlos Diz-Gómez, Silvia Varela

**Affiliations:** 1 Well-Move Research Group, Galicia-Sur Health Research Institute (IIS Galicia Sur), SERGAS-UVIGO, 36310 Vigo, Spain; 2 Department of Special Didactics, University of Vigo, 36310 Vigo, Spain

**Keywords:** child, energy metabolism, exercise, pediatric obesity, testing

## Abstract

This study aims to investigate the potential relationship between energy expenditure, physical fitness level, physical activity, and body mass index among children taking part in a 30 min school recess. A total of 259 participants from three schools were included in this study. Data on energy expenditure during recess, age, gender, anthropometric measurements, global physical fitness, and physical activity index were recorded. The evaluation sessions occurred twice a week on alternate days over two weeks. A significant gender difference was observed in energy expenditure during recess, favoring boys (*p* < 0.01). The participants classified as very active exhibited significantly higher scores compared to those categorized as sedentary and moderately active, respectively (*p* < 0.01), with a poor but significant correlation (rho: 0.208; *p* < 0.001). There were significant differences between energy expenditure and cardiorespiratory fitness and energy expenditure and global physical fitness (*p* < 0.01) with fair (rho: 0.289; *p* < 0.001) and poor (rho: 0.196, *p* = 0.001) correlation, respectively. In contrast, there were no significant differences by body mass index categories (*p* = 0.571). These results suggest that gender, physical activity index, and global physical fitness were found to influence energy expenditure during a 30 min recess. However, no significant relationships were found with the body mass index.

## 1. Introduction

In the era of technology and constant connectivity, children are facing a growing epidemic of physical inactivity [1]. Studies have indicated that the prevalence of screen time is rising. For instance, the NHANES study found that 66% of children spent 2 or more hours sitting watching TV/videos and around 56% spent 2 or more hours of their leisure time using a computer. These findings suggest a consistent decrease in children’s physical activity levels over the last 15 years [2]. Indeed, industrialization and digitalization are deemed to be responsible for a significant decrease in the performance of physical activity, especially among children, who barely reach the physical activity (PA) levels prescribed by the American College of Sports Medicine or the World Health Organization [3].

This lack of regular exercise can contribute to various issues, including an elevated risk of injuries [4] and obesity [5], which is associated with different comorbidities, such as diabetes mellitus type II, cardiovascular diseases, and cancer [6]. Furthermore, regular physical activity (PA) among children not only promotes better physical development but also improved cognitive and emotional performance, as well as increased academic performance [7].

Despite the numerous advantages associated with an active lifestyle, sedentary behaviors remain highly prevalent among children [8], and this issue is compounded by the sedentary nature of many school activities [9]. This lack of physical activity has indirectly burdened public health, as a child who is physically inactive is more likely to grow into an inactive adult [3]. Thus, efforts to increase PA levels are crucial, especially during early adolescence, a phase during which declines in PA prevalence begin to be noticeable [10].

In a context where technological advancements have led to an increased sedentary behavior, a model of physical activity transition has emerged. Strategies to enhance physical activity patterns are crucially needed, especially among children [11]. Considering the substantial time spent by children at school, this environment has been recognized as a pivotal opportunity for them to increase their PA levels [12]. In this regard, physical education classes and recess time are arguably the most natural opportunities for children to accumulate PA time during the school day [13].

Indeed, school recess present an excellent opportunity to promote PA among children, and their behavior during this period has been the subject of numerous research studies, focused on different variables such as obesity, PA prevalence, sex, and PA intensity [14]. For instance, Stratton et al. [15] demonstrated that normal-weight boys were significantly more active than overweight boys. In the study conducted by Chen et al. [16], they found a significant association between PA during recess and children’s physical fitness and total weekly PA. Another well-known fact is that boys are consistently more active than girls [17,18], and notable disparities exist in the intensity of PA during school recess [19].

Recently, there has been an acknowledgment of the need to enhance the quality of school recess time. Various strategies have emerged, including improvements in equipment and the overall environment, all with the shared objective of increasing children’s energy expenditure [20].

A number of studies have been focused on energy expenditure, with findings indicating that recess represented 8.7% of the daily PA energy expenditure during school days [21]. Despite significant progress in this field, there are still several aspects that remain under-researched. For instance, while some studies have suggested a correlation between children’s body mass index (BMI) and energy expenditure during recess [22], other studies have produced conflicting results regarding this relationship [23]. This is a matter of concern, as there is a need to increase recreational PA levels among overweight or obese school-children [24], and school break time should be a golden opportunity for achieving this goal. Thus, there is a need for scientific evidence confirming whether BMI impacts energy expenditure during school break time, in order to develop strategies aimed at enhancing PA levels in this population.

Similarly, the impact of physical fitness and PA on energy expenditure during recess remains unclear, which hinders the establishment of connections between energy expenditure during recess and various health-related variables. Researching fitness among children and, particularly, identifying existing gaps in the literature are a must, as fitness is considered a strong marker of health [25]. In this context, there has been a recognized need for a better understanding of weekly PA composition, especially during school time [26]. Therefore, it is equally important to identify whether children with lower weekly PA levels are also the ones exhibiting more sedentary behavior during recess. It could be the case that lower physical fitness and reduced PA levels contribute to a diminished energy expenditure. Therefore, in addition to enhancing equipment and the environment in which recess takes place, efforts aimed at transforming this part of the school schedule into an ideal context for increasing physical activity levels should particularly consider this subgroup as a target. Nevertheless, existing studies on the impact of fitness and PA levels on energy expenditure during recess are scarce. Gaining a deeper understanding of these relationships holds immense value, as it would aid in developing effective strategies for promoting PA during recess and enhancing the overall health among children.

Therefore, this study aimed to explore whether energy expenditure during recess is modulated by BMI, physical fitness, and PA levels among children.

## 2. Materials and Methods

### 2.1. Participants

The participants were healthy rural and urban children who were recruited from three different schools in the north of Spain. The schools were located in three different municipalities, with a population size ranging from 25,000 to 35,000 inhabitants. All the schools had open-air dirt playgrounds and an asphalt futsal court measuring 20 m × 40 m. The inclusion criteria for participation were (a) being in the 5th or 6th year of primary education and (b) having no medical condition that could hinder performance of the activities proposed in the research. Children who showed intellectual or physical disabilities that prevented them from understanding the test protocol or performing the tests correctly were excluded from the research. Written informed consent was received from the parents or guardians of all the children who took part in the research study. The study design was approved by the Ethics Committee of the Faculty of Education and Sports Science (University of Vigo) with code 04-1421.

### 2.2. Measurements

#### 2.2.1. Energy Expenditure

The energy expended during recess was measured using a Fitbit wristband, a device which has been previously used in this type of study [27]. This device estimates PA variables such as steps taken, energy expenditure, and active minutes via accelerometers and optical plethysmography in 30 s epochs. The “normal” setting was chosen as the default setting. The measures of interest in the study were the total time and kilocalories expended during recess. The data were synchronized to a mobile phone each day once the recess sessions were over.

#### 2.2.2. Anthropometry

Weight (kg) and height (cm) were measured without shoes and with light clothing by means of a Tefal digital scale (type PP1200VO) and a field stadiometer (Seca 220). Each child’s BMI was calculated using the following formula: body mass/height^2^ (kg/m^2^). The subjects were classified according to the BMI-specific cut-off points previously established in the growth chart of Fernández et al. [28].

#### 2.2.3. Physical Fitness

Three field-based fitness tests from the Eurofit battery [29] were used to assess the physical fitness of the children [30].

Cardiorespiratory fitness was assessed using the Course Navette test [31]. The participants were previously familiarized with this test, as it is usually performed by physical education teachers. The test consists of running back and forth on a 20 m track marked between two separate lines for as long as possible. The rhythm is set using audio signals. The initial speed is 8.5 km/h and is increased by 0.5 km/h intervals every 1 min. The subjects must step behind the 20 m line when the audio signal or beep is heard. The test finishes when the subject stops because of fatigue or fails to reach the end line concurrent with the beep on two consecutive occasions. This test was conducted in groups of ten children, and its performance was recorded by counting the number of 20 m laps (one lap = 20 m) and measuring the total time (in seconds). The participants were encouraged by the physical education teacher to perform the test until reaching maximal exhaustion. The Course Navette has shown good criterion-related validity (r = 0.78, 0.72–0.85) [32] and reliability (ICC = 0.78 to 0.93) in children [33].

Handgrip strength was assessed by means of a handgrip dynamometer to be squeezed with as full a force as possible with the strongest arm fully extended slightly away from the body and one’s palm facing inward. To exert pressure, only the fingers of the assessed hand were instructed to flex. The best of two attempts was recorded. This gold-standard strength measure has demonstrated a high reliability (ICC = 0.95) among children [34].

Flexibility was measured using the Sit-and-Reach (SR) test [35]. The children were required to sit on the floor without shoes, with their knees straight, and their feet placed flat against the front-end panel of a box (45 × 32 × 35 cm), which had a measuring scale inscribed in its top. Then, they were asked to slowly reach forward as far as possible while placing their palms down along the measuring scale and hold the position for approximately two seconds. The most distant point reached with the fingertips was recorded. The best of two trials was retained for analysis. The SR has yielded moderate-to-good validity (r = 0.66–0.86) and high reliability (ICC > 0.90) coefficients among scholars [36].

Three senior students of the Degree in Primary Education, familiar with the test protocols, administered the three field-based fitness tests during physical education lessons. The physical education teachers at each school oversaw the assessment of the session performance.

The Z-scores of the absolute handgrip strength, Course Navette tests, and SR tests, stratified by age and sex, were calculated as follows: Z-score = (raw score − sample mean)/sample standard deviation. An overall physical fitness Z-score was then calculated as the mean of the standardized scores of each physical fitness component. We used these Z-scores to divide the children into two categories, depending on whether they performed below (z < 0) or over (z > 0) the reference mean.

#### 2.2.4. Physical Activity

The Spanish version of the Assessment of Physical Activity Levels Questionnaire (APALQ) [37] was administered during the physical education classes. The questionnaire consisted of five questions, each with four specific options. The responses were measured on a 4-point Likert scale, ranging from 1 (lowest value) to 4 (highest value). However, for questions 3 and 4, a different scoring system was utilized, with scores ranging from 1 to 5 points. To assess the children’s activity levels, a physical activity index (PAI) was utilized, which had a maximum score of 22 points (the sum of the maximum scores from each question in the APALQ). Three levels of PAI were considered: inactive (5–10 points), moderately active (11–16 points), and highly active (≥17 points) [38]. The APALQ has shown a moderate criterion validity (r = 0.47) and a good reliability (ICC: 0.74–0.77) among Spanish children [37].

### 2.3. Procedure

The protocol consisted of measuring the participants’ energy expenditure during school recess time by means of Fitbit Charge 4TM (Fitbit, Inc., San Francisco, CA, USA). For this purpose, on the day the measurement was scheduled, each child was given a bracelet in the classroom, was explained how it worked, and programmed it to measure energy expenditure in outdoor activities. After 30 min, all the wristbands were paused, and the energy expenditure in kcals of each person was noted. During the recess, the participants were not encouraged to do any kind of PA. Moreover, it was emphasized that it was important that they did no more and no less than what they usually did. For each participant, energy expenditure was assessed during two non-consecutive recesses over a two-week period. A total of 25 children were evaluated in each recess. The data on energy expenditure were registered during the spring on non-rainy days, with temperatures approximately ranging between 11 and 15 °C. Data on anthropometry, physical fitness, and PA were collected during the first week of this research.

### 2.4. Statistical Analysis

The statistics were computed using the Statistical Package for the Social Sciences (SPSS v24, Armonk, NY, USA: IBM Corp.) The data are presented as mean ± standard deviation (SD) for normally distributed variables. The Kolmogorov–Smirnov test was performed to check for normality of distribution. We compared energy expenditure with other qualitative variables with *t*-Student test or analysis of variance, as appropriate. It was found that the BMI, cardiorespiratory fitness (Course Navette periods), and the physical activity index (PAI, calculated with the APALQ) exhibited a non-normal distribution. Because of this, Spearman’s rank correlation was used to examine the association between variables as well as the possibility of non-linear correlations. The correlation values were characterized as follows: poor, 0.00–0.19; fair, 0.20–0.39; moderate, 0.40–0.59; good, 0.60–0.79; and high, 0.80–1 [39]. The level of significance was set at a *p*-value less than 0.05.

## 3. Results

All the participants completed the protocol and final testing; there were no dropouts. The total number of assessments performed using the Fitbit was 518. Consequently, a total of 259 (mean ± SD: age, 11.40 ± 0.63 years; height, 148 ± 8 cm; body mass, 43.47 ± 10.84 kg) healthy children were included in the analysis of this study. The final sample consisted of 44.4% girls, providing a balanced representation of both genders. Table 1 shows the main characteristics of the sample.

Table 2 shows the mean values obtained by the boys and by the girls. Significant differences in energy expenditure were observed, favoring the boys (141.57 ± 25.92 vs. 123.48 ± 21.61 kcal; *p* < 0.01). The participants considered to be “very active” showed significantly higher scores than those considered to be “sedentary” and “moderately active”, respectively (139.77 ± 24.9 vs. 129.13 ± 26.12 and 129.17 ± 25.27 kcal; *p* < 0.01) (Table 3). There were no significant differences by BMI categories (*p* = 0.571). However, the results showed that underweight children achieved the highest results (137.17 ± 24.6 kcal), followed by those within the normal weight (133.96 ± 25.94 kcal) and obese ranges (133.86 ± 33.09 kcal), and the lowest scores were observed among the overweight children (129.1 ± 23.73 kcal). There were significant differences between mean energy expenditure and cardiorespiratory fitness-Z and physical fitness-Z (*p* < 0.01). The subjects with above-average fitness had higher caloric expenditure values.

The influence of BMI, PA, and physical fitness on energy expenditure is shown in Table 4. Poor significant correlations were observed between energy expenditure and PAI for the entire sample (rho: 0.208; *p* < 0.001). Similar correlations were found for the boys (rho: 0.175; *p* = 0.036) but not for the girls (rho: 0.114, *p* = 0.225). Energy expenditure and BMI were not significantly correlated in our sample (rho: −0.086, *p* = 0.167). Significant poor associations were also noted between mean energy expenditure and physical fitness-Z for the entire sample (rho: 0.196, *p* = 0.001) and for the boys (rho: 0.289, *p* < 0.001) but not for the girls (rho: 0.087, *p* = 0.357). Fair significant associations were also noted between mean energy expenditure and cardiorespiratory fitness-Z (rho: 0.289; *p* < 0.001) when the sample was analyzed as a whole.

## 4. Discussion

There is a lack of scientific evidence regarding the impact of physical fitness and PAI on energy expenditure, particularly in the recess school context. This study aimed to address the relationship between energy expenditure and physical fitness level, PAI, and BMI in primary school children during recess. The findings obtained in this study can provide valuable insights and guidance for physical education teachers in selecting appropriate strategies to promote PA during recess and enhance the overall health of the children. Additionally, researchers in the field of physical education can benefit from this study’s contribution to the existing literature on energy expenditure during recess.

One main finding of interest in this research is that the mean energy expenditure was 133.54 kcal during a 30 min recess. These results closely align with the findings of Howe et al. [23] who suggested that engaging in enjoyable and energy-demanding games during recess can lead to an average expenditure of approximately 100 kcal for most children during a 30 min recess. A recent study yielded lower values (around 75–82 kcal), although energy expenditure was estimated using secondary data obtained from US elementary schools (recess total time 26.5 min) [20].

When analyzed by gender, the energy expenditure was higher in the boys than in the girls during a 30 min recess (141.57 ± 25.92 vs. 123.48 ± 21.61 kcal, respectively). This finding is consistent with a study conducted by Hall-Lopez et al. [40], which also reported a significant gender-based difference in energy expenditure. Additionally, another study conducted by Hall-López & Ochoa-Martínez [41] reported a significant difference in energy expenditure based on gender in children with a normal percentage of fat during a 30 min recess. However, the kcal consumption was lower in the above-mentioned study (boys: 94.2 ± 14.2, girls: 87.35 ± 15.47 kcal) compared to our results. This difference in kcal consumption could be attributed to either the age disparity between the samples [42] or the playground’s characteristics [43]. The higher energy expenditure observed in boys during recess could be attributed to previously reported findings indicating that boys consistently spend more time engaging in moderate-to-vigorous physical activities than girls during school recess, irrespective of whether the activities are pre-planned by teachers or free [44]. These results are consistent with recent research indicating that boys spend significantly more minutes in the moderate-to-vigorous portion of physical activity than girls during a typical week [45]. The main reasons behind these sex differences have been previously discussed [46]. It might be that, during recess, boys usually engage in moderate and vigorous physical activity mainly by playing competitive sports, while girls perform lighter activities, such as climbing or balancing. Additionally, the prevalence of areas designed for ball sports, which are mainly practiced by boys, leaves limited options for girls to engage in their preferred physical activities.

The relationship between body composition and school recess in children is a topic of interest. For instance, Thalken et al. [47] indicated that policies related to recess access (i.e., total time, equipment characteristics, and withholding recess for behavioral or academic reasons) were significantly associated with children’s BMI. In this regard, it could be expected that overweight/obese children would exhibit more sedentary behaviors, as they may have low fitness and motor skill abilities, making it difficult to engage in activities of a high intensity [48]. However, in the present research, no significant differences in energy expenditure based on BMI categories were identified. In relation to this, no clear pattern seems to emerge. For instance, Howe et al. [23] and Bartholomew et al. [49] reported similar results to ours. On the contrary, Latorre-Román et al. [22] observed a significant correlation between BMI and energy expenditure in primary children. Notably, in their research, the boys who were overweight or obese exhibited high energy expenditures. In our study, although the highest energy expenditure was observed in underweight children, obese students showed almost the same energy expenditure as students with a normal weight. Similarly, Sulla et al. [50] reported a lack of significant differences between normal-weight and overweight boys and girls aged 10–13 years regarding their PA levels during recess. Finally, Carriedo et al. [48] found that children with a normal weight showed a statistically significantly higher energy expenditure in comparison with overweight, obese, and underweight children. Interestingly, BMI was not associated with sedentary behavior. Taken together, these findings emphasize previous indications suggesting that the causal connection between BMI and PA behavior among elementary children is somewhat contentious [45]. Nonetheless, it is important to interpret these results with caution due to the high prevalence of children with a normal weight, which might have influenced the outcomes. Future research should consider this aspect when examining energy expenditure during unstructured 30 min breaks in primary school children.

In this study, we also analyzed the impact of physical fitness on energy expenditure, which is an under-researched topic. In a seminal study, aerobic fitness and muscular strength were significantly associated with children being physically active during recess [16]. We did not find significant differences concerning handgrip strength and flexibility in relation to children’s energy expenditure. However, the results suggest that children with higher levels of cardiorespiratory and global physical fitness exhibited increased energy expenditure during a 30 min recess. These findings align with those reported by Bartholomew et al. (2022), who found that aerobic fitness was significantly and positively linked to moderate-to-vigorous PA during recess. Similarly, Calahorro et al. [51] indicated that sedentary time during recess was inversely and significantly associated with cardiorespiratory fitness. Finally, López et al. [52] reported that a higher intensity of PA school recesses was positively associated with global physical fitness. Other authors have suggested that fitness does not affect recess PA levels, but their results are strongly limited since the data on both variables were self-reported [53]. In conclusion, our results suggest that high CRF levels allow for the involvement in high-intensity activities, resulting in greater energy expenditures.

Another noteworthy finding is that PAI has a significant effect on energy expenditure. In our research, the children who were considered to be “very active” showed significantly higher scores than the “sedentary” and “moderately active” children in energy expenditure. To our knowledge, few investigations have analyzed this relationship during recess in primary school children. Similar to our findings, Gonzalez-Suarez & Grimmer-Somers [54] observed that self-assessed PA showed a modest correlation (r^2^ = 0.21) with the amount of PA performed during school days among prepubescent children. On the other hand, Ariz et al. [46] did not report any significant association between PA outside school and during recess, while Long et al. [55] suggested that a higher number of minutes spent performing moderate-to-vigorous PA at school was associated with a higher total time of daily moderate-to-vigorous PA among youth aged 6–19 years. These results highlight the importance of promoting regular PA, particularly during school recess, to improve children’s PA habits outside school.

This manuscript provides novel data for the literature on this topic as it examines the effects of physical fitness and PAI on energy expenditure during recess in primary school children. The practical implications drawn from this research include the confirmation that recess contributes to increased energy expenditure among primary children. As a result, common practices such as withholding recess as a sanction or for academic purposes [56] should be avoided. Similarly, school recess should last at least 30 min, in contraposition to current recommendations of 20 min [57]. Finally, efforts to enhance energy expenditure during recess should be targeted more towards children with lower levels of physical fitness rather than solely focusing on overweight or obese children.

Nonetheless, some limitations have to be addressed. First, the PAI was self-reported by the children and not through objective methods such as accelerometers. The limitation of self-reported measures include the potential for recall bias, where accurate recollection of activity levels may be difficult, along with challenges in interpreting the questions. Secondly, the low prevalence of high BMI in the study participants could have influenced the results. Another limitation of the current study was the use of field tests to assess cardiorespiratory fitness. It should also be acknowledged that we did not account for whether the children had participated in physical education sessions before the school recess. This factor could have influenced the recorded energy expenditure. Finally, the generalizability of these recess data may be questioned because of the recess environment in the study schools (located in Northern Spain). Given this limitation, particularly concerning the seasonality comparisons, it would be prudent to exercise caution before extrapolating our conclusions to geographical areas with milder climates.

In conclusion, our study showed that energy expenditure during recess was related to gender, PAI, and global physical fitness. However, no significant relationships were found with BMI. The current data highlight the importance of the promotion of PA during school recesses to favor possible improvement in the health status of primary school children. Future studies with larger sample sizes, extended monitoring over a longer school period, and including objectively measured weekly physical activity are needed to confirm these findings.

## 5. Conclusions

This study showed that energy expenditure during recess was related to gender, PAI, cardiorespiratory fitness, and global physical fitness. However, no significant relationships were found with BMI. The current data highlight the importance of the promotion of PA during school recesses to favor possible improvements in the health status of primary school children.

## Figures and Tables

**Table 1:** Demographic characteristics of the participants (n = 259).

		n (%)	$\bar{X}$ ± SD	Range
Gender	Boys	144 (55.6)		
	Girls	115 (44.4)		
Age (years)		259	11.40 ± 0.63	10.1–13.3
	10	84 (32.4)		
	11	123 (47.5)		
	12	52 (20.1)		
School year	5th	129 (49.8)		
	6th	130 (50.2)		
Body mass (kg)		259	43.47 ± 10.84	22.6–92
Height (cm)		259	148 ± 8	122–169
BMI (kg/m^2^)		259	19.59 ± 3.86	12.97–41.44
BMI categories	Underweight	33 (12.7)		
	Normal range	171 (66)		
	Overweight	44 (17)		
	Obese	11 (4.2)		
Physical fitness	Cardiorespiratory fitness (periods)	259	3.95 ± 2.29	0.5–12.5
	Handgrip strength (kg)	259	16.62 ± 4.42	7.1–36.5
	Flexibility (cm)	259	17.09 ± 7.44	1–35
PAI		259	15.14 ± 3.75	5–22
Energy expenditure (Kcal)		259	133.54 ± 25.69	74–231

Abbreviations: BMI: body mass index; PAI = physical activity index; SD = standard deviation; and $\bar{X}$ = mean.

**Table 2:** Mean values comparison between boys and girls (n = 259).

		Boys				Girls			
		N	$\%/\bar{X}$	SD	Range	N	$\%/\bar{X}$	SD	Range
Age (years)		144	11.38	0.65	10.1–13.2	115	11.42	0.59	10.4–13.3
	10	51	35.4			33	28.7		
	11	63	43.8			60	52.2		
	12	30	20.8			22	19.1		
Body mass (kg)		144	43.37	10.88	26.6–92	115	43.59	10.83	22.6–85.1
Height (cm)		144	148	7	133–169	115	149	9	122–167
BMI categories		144	19.68	3.99	13.69–41.44	115	19.48	3.70	12.97–31.26
	Underweight	16	11.1			17	14.8		
	Normal range	95	66			76	66.1		
	Overweight	26	18.1			18	15.7		
	Obese	7	4.9			4	3.5		
Physical fitness									
	Cardiorespiratory fitness (periods)	144	4.43	2.29	0.5–9.5	115	3.36	2.16	0.5–12.5
	Handgrip strength (kg)	144	16.46	4.22	7.3–36.5	115	16.83	4.68	7.1–29.1
	Flexibility (cm)	144	15.15	6.62	1–31	115	19.52	7.72	1–35
PAI categories		144	15.72	3.80	5	22	115	3.56	6–21
	Sedentary	18	12.5				21		
	Moderately active	53	36.5				60		
	Very active	73	50.7				34		
Energy expenditure (Kcal)									
	Recess 1	144	143.69	30.54	88–231	115	124.53	22.94	86–211
	Recess 2	144	139.46	30.74	74–228	115	122.43	29.64	76–221

Abbreviations: BMI: body mass index; PAI = physical activity index; SD = standard deviation; and $\bar{X}$ = mean.

**Table 3:** Significant correlates of energy expenditure (kcal) in a single-variable analysis (n = 259).

		$\bar{X}$ ± SD	*p*
Gender			<0.001 *
	Boys (n = 144)	141.57 ± 25.92	
	Girls (n = 115)	123.48 ± 21.61	
Age (years)			0.642
	10 (n = 84)	132.46 ± 23.12	
	11 (n = 123)	135.09 ± 27.18	
	12 (n = 52)	131.61 ± 26.24	
BMI categories			0.571
	Underweight (n = 33)	137.17 ± 24.6	
	Normal range (n = 171)	133.96 ± 25.94	
	Overweight (n = 44)	129.1 ± 23.73	
	Obese (n = 11)	133.86 ± 33.09	
PAI categories			0.004 *
	Sedentary (n = 39)	129.13 ± 26.12	
	Moderately active (n = 113)	129.17 ± 25.27	
	Very active (n = 107)	139.77 ± 24.9	
Physical fitness-Z			<0.001 *
	Higher than average (n = 125)	139.4 ± 27.47	
	Lower than average (n = 134)	128.07 ± 22.67	

Abbreviations: BMI: body mass index; PAI = physical activity index; SD = standard deviation; and $\bar{X}$ = mean. Independent sample *t*-tests were used for gender and physical fitness-Z comparisons, while an analysis of variance was used for all other comparisons. * Denotes a significant correlation (*p* < 0.05).

**Table 4:** Correlations with the energy expenditure variable.

	Total n = 259		Boys n = 144		Girls n = 115	
	Spearman		Spearman		Spearman	
	Rho	*p*	Rho	*p*	Rho	*p*
Age (years)	0.000	1.000	0.048	0.571	−0.052	0.584
Body mass (kg)	−0.082	0.190	−0.068	0.416	−0.108	0.250
Height (cm)	−0.062	0.320	−0.094	0.264	0.018	0.853
BMI (kg/m^2^)	−0.086	0.167	−0.063	0.453	−0.148	0.115
PAI (mean)	0.208 *	0.001 *	0.175 *	0.036 *	0.114	0.225
Cardiorespiratory fitness (periods)	0.386 *	<0.001 *	0.327 *	<0.001 *	0.259 *	0.005 *
Handgrip strength (kg)	−0.012	0.852	0.056	0.505	−0.071	0.451
Flexibility (cm)	−0.017	0.785	0.190 *	0.022 *	−0.009	0.927
Cardiorespiratory fitness-Z (by age and gender)	0.289 *	<0.001 *	0.318 *	<0.001 *	0.250 *	0.007 *
Handgrip strength-Z (by age and gender)	−0.004	0.945	0.050	0.553	−0.079	0.404
Flexibility-Z (by age and gender)	0.092	0.142	0.188 *	0.024 *	−0.015	0.870
Physical fitness-Z (by age and gender)	0.196 *	0.001 *	0.289 *	<0.001 *	0.087	0.357

Abbreviations: BMI = body mass index; and PAI = physical activity index. * Denotes a significant correlation (*p* < 0.05).

## Data Availability

Data are contained within the article.
